# Change in Ubiquitin Proteasome System of Grass Carp *Ctenopharyngodon idellus* Reared in the Different Stocking Densities

**DOI:** 10.3389/fphys.2018.00837

**Published:** 2018-07-05

**Authors:** Yiqing Sun, Xiao Liang, Jie Chen, Rong Tang, Li Li, Dapeng Li

**Affiliations:** Hubei Provincial Engineering Laboratory for Pond Aquaculture, National Demonstration Center for Experimental Aquaculture Education, College of Fisheries, Huazhong Agricultural University, Wuhan, China

**Keywords:** ubiquitin proteasome system, protein metabolism, growth, fish, crowding stress

## Abstract

The ubiquitin proteasome system (UPS) is a highly regulated mechanism of intracellular protein degradation and turnover. To evaluate the effect of crowding stress on the UPS in fish, grass carp (*Ctenopharyngodon idellus*) were randomly reared at low stocking density (LSD, 0.9 kg m^−2^) or high stocking density (HSD, 5.9 kg m^−2^) for 70 days. The expression of the genes regulating UPS, stress-related parameters, and profiles of amino acid in white muscle as well as growth rate of fish reared at two stocking densities were investigated. Fish exhibited significantly higher growth rate in the LSD group compared to the HSD group. Serum concentrations of cortisol, total protein, and glucose did not vary significantly in fish between two groups. There was no significant difference in the mRNA levels of *nrf2*, *keap1*, and *hsp90* in white muscle of fish stocked at two densities at the endpoint of the experiment. In the UPS pathway, the expressions of *ub*, *chip*, *psmc1* in the LSD group were significantly higher than those in the HSD group (*P* < 0.05). Ubiquitinated protein level and the content of 3-Methylhistidine elevated significantly in the LSD group (*P* < 0.05). The mRNA levels of *mafbx*, *murf1*, and *s6k1* in the LSD group were significantly higher than those in HSD group (*P* < 0.05). These results illustrate that the fish cultured in lower stocking density would exhibit a greater growth rate and a fast protein turnover in muscle.

## Introduction

The ubiquitin proteasome system (UPS) is important for regulating protein degradation and function (Pickart and Eddins, 2004). The function of UPS is involved in various cellular processes, such as signal transduction, metabolic regulation, cell cycle control, development, apoptosis, protein quality control, and antigen presentation (Glickman and Ciechanover, 2002; Flick and Kaiser, 2012; Sommer and Wolf, 2014). The conjugation process of ubiquitinylation is a complex reaction that requires ubiquitin (*ub*), ubiquitin activating enzyme (E1), ubiquitin conjugating enzyme (E2), and ubiquitin protein ligase (E3) (Romn and Reznick, 2016). E3 ligases have a central role in coupling ubiquitin to the targeted protein and generate the specificity of the mechanism (Jackson and Eldridge, 2002). The UPS is an ATP-dependent proteolytic system and involves two main steps. First, targeted protein is polyubiquitinated with the specificity determined by the E3 ubiquitin ligase enzyme. Second, the ubiquitinated protein is recognized by 26S proteasome which degrades the substrate into peptides (Ciechanover, 1994).

Muscle RING finger 1 (MuRF1) and muscle atrophy Fbox (MAFbx/atrogin-1) have been regarded as central markers involved in protein degradation (Bodine et al., 2001). These two proteins, belong to ubiquitin E3 ligases, consistently highly express in atrophying muscle caused by cancer, diabetes, fasting, and limb immobilization (Sacheck et al., 2007). The growth of skeletal muscle depends on the balance between protein synthesis and degradation in teleost fish as well as other vertebrates. UPS is a major system for protein degradation in eukaryotic cells (Pickart and Eddins, 2004), and regulate the turnover of protein in rainbow trout (*Oncorhynchus mykiss*) (Cleveland and Evenhuis, 2010). A study revealed that specific growth rate was negatively correlated with 20S proteasome activity in the liver of rainbow trout (Dobly et al., 2004). Consequently, fish growth rate probably be related to the activity of UPS. As far as anabolism is concerned, mTOR (mammalian/mechanistic target of rapamycin complex) and S6K1 (ribosomal protein S6 kinase 1) are of importance in regulating protein synthesis and controlling cell growth (Huynh et al., 2017). The expression levels of MAFbx, MuRF1, and mTOR elevated significantly as IGF-1 increased in animals (Liu H.H. et al., 2014). It is implied that the growth enhancement is related to gene expression of mTOR, MAFbx, and MuRF1.

There are many external factors affecting growth, such as stocking density, food nutrition, temperature, and water quality (Siikavuopio and Sæther, 2006; Fraser and Rogers, 2007; Valenzuela et al., 2017). For example, previous study suggested that Nile tilapia (*Oreochromis niloticus*) obtained successful growth, maximum fertilization, and survival rate of fry up to 15% salinity (Malik et al., 2018). Also, Rahim et al. (2017) reported that good performances of black fin sea bream (*Acanthopagrus berda*) were closely associated with factors including food nutrition, temperature, and salinity. With the development of intensive aquaculture, fish reared at high stocking density exhibited reduced growth rate with lower resistance to diseases (Benjamín et al., 2008; VargasChacoff et al., 2011). In addition, high stocking density exerted negative effect on food conversion efficiency in fish (Beveridge et al., 1997). Excessive high stocking density often causes crowding stress and impairs fish welfare. Under the condition of environmental stress, the UPS can be activated to maintain cellular protein homeostasis, regulating a dynamic balance in protein synthesis and degradation processes. Especially, the ubiquitin ligases play important role in stress sensing, signaling, and activation of the hypoxia response pathway (Flick and Kaiser, 2012). Thermal stress resulted in a significant elevation of 20S proteasome activity in the liver and gill of notothenioid fishes (Todgham et al., 2017). And the downregulation of protein ubiquitination pathway was demonstrated in the liver of rainbow trout exposed to crowding stress (Rebl et al., 2017). *Nrf2* is a master transcription factor of the cellular antioxidant response through *keap1*-*nrf2* signaling pathway (Dodson et al., 2015). The misfolded or unfolded proteins during stress are refolded by chaperone systems first, when refolding is unsuccessful the UPS degrades proteins (Flick and Kaiser, 2012). HSP70 and HSP90 play an important roles in both refolding and selection of proteins for UPS through CHIP ubiquitin ligase (Benarroch, 2011).

**FIGURE 1:**
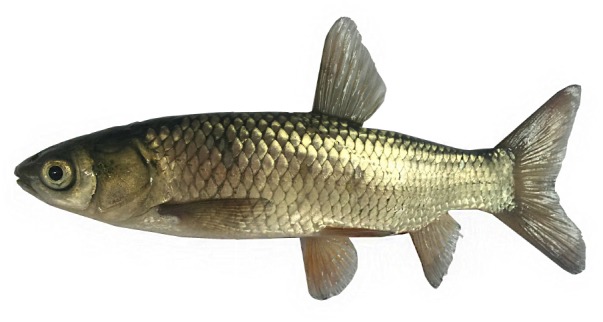
Grass carp, *Ctenopharyngodon idellus* (Photo by Dapeng Li).

To date, little is known about the response of UPS to crowding stress in fish. Grass carp (*Ctenopharyngodon idellus*) (Cypriniformes, Cyprinidae, Leuciscinae) (**Figure 1**) is a typical herbivorous fish, and the most popular cultured fish species in China (Jin et al., 2013). The aquaculture production of grass carp ranks top among the world’s freshwater aquaculture industries now (Xie et al., 2018). Its production reached more than 5 million tons and supplied nearly 20% of the total freshwater fish production in China (FAO Yearbook, 2016). The purpose of this study was to investigate the activity of UPS in fish reared at different stocking densities and to elucidate the relationship between crowding stress and muscular protein metabolism mediated by UPS.

## Materials and Methods

### Fish and Experimental Design

All fish were collected from a fish farm in Hubei province, China. Before the start of the experiment, fish were reared in the freshwater tanks. We randomly distributed 140 individuals (initial body weight: 98.48 ± 6.00 g) into eight circular fiberglass tanks at two stocking densities (four tanks in each stocking density group): low stocking density (LSD, 0.9 kg m^−2^, 5 fishes per tank), and high stocking density (HSD, 5.9 kg m^−2^, 30 fishes per tank) for a period of 70 days. Water temperature (20 ± 0.7°C), pH (7.5 ± 0.3), dissolved oxygen (above 7.0 mg L^−1^), and ammonia concentration (less than 0.02 mg L^−1^) were maintained at the same water quality level in each group.

To gain an insight into the relation between stocking density and growth, we examined the body weight, weight gain rate (WGR), specific growth rate (SGR), feed conversion efficiency (FCR), and feed ration (FR) in each group during the experimental period. The growth performances were measured with eight fish (two fish per tank).

$$\begin{array}{l} \text{WGR}=100\times (\text{Wt}-\text{Wo})/\text{Wo}\\ \text{SGR}=100\times (\text{LnWt}-\text{LnWo})/\text{T}\\ \text{FCR}=\text{FI}/(\text{Wt}-\text{Wo})\\ \text{FR}=100\times \text{FI}/[0.5(\text{Wt}+\text{Wo})\times \text{T}] \end{array}$$

Wo, initial weight (g); Wt, final weight (g); T, feeding days; FI, feed intake (dry weight).

At the end of the experiment, fishes were randomly selected from each group (5 individuals per tank) and anesthetized with tricaine methanesulfonate (MS-222, 200 mg/L). Dorsal white muscle and blood of fish were collected for analysis. Muscle samples were immediately frozen in liquid nitrogen, and then stored at −80°C until RNA extraction. Each mix received from five fish acted as one sample used for subsequent experiment.

This study was approved by the Institutional Animal Care and Use Committees (IACUC) of Huazhong Agricultural University (HZAU) (Wuhan, China). The experiment was made according to the reporting of *In Vivo* Experiments (ARRIVE) guidelines and “Guidelines for Experimental Animals” of the Ministry of Science and Technology (Beijing, China). All efforts were made to minimize suffering of sampled fishes.

### Measurement of Serum Hormones and Biochemistry

Serum biochemistry was analyzed to determine the level of fish stress. Serum cortisol level was analyzed using a commercial RIA kit (Coated Tube Cortisol ^125^I RIA Kit, BNIBT, and Beijing, China) according to the manufacturer’s instruction. Serum glucose (GLU) and total protein (TP) were measured by a commercial kit (Sysmex Wuxi Co., Let., China).

### RNA Extraction

The RNA was extracted from 80 mg of muscle, homogenized by RNA isolater (Vazyme). RNA integrity was detected in 1.1% agarose gel electrophoresis, and the concentration was quantified spectrophotometrically. For each sample, 1 μg of total RNA was reverse transcribed in the presence of oligo-dT primers using a PrimeScript RT reagent Kit with gDNA Eraser (Perfect Real Time) (Takara, Dalian, China) according to the manufacturer’s instructions.

### Molecular Cloning

The 20S proteasome subunit alpha type 2 (*psma2*) was chosen as candidate gene representing an α-subunit, respectively. And 26S proteasome subunit ATPase 1 (*psmc1*) was chosen as a representative ATPase subunit in the 19S regulatory particle (Lupatsch et al., 2010). We searched for and downloaded *ubiquitin* (*ub*), *murf1*, *mafbx*, *chip*, *nrf2*, *keap1*, *psma2*, *psmc1*, *s6k1*, *mtor*, *hsp70*, and *hsp90* genes’ sequences from National Center for Biotechnology Information (NCBI) databases. Primers were designed by Primer Premier 5. Sequences were initially analyzed using Blast search to confirm the correct genes were cloned.

### Real-Time qRT-PCR

Quantitative real-time (qRT)-PCR was performed on a Quant Studio 6 Flex Real-Time PCR Detection System (Applied Biosystems, United States). The 20 μL reaction mixture contained 10 μl Hieff^TM^ qPCR SYBR^®^ Green Master Mix (YEASEN, Sanghai, China), 0.4 μL of each of the specific primers, 7.2 μL RNase-free water, and 2 μL cDNA template. The gene-specific primers (**Table 1**) were designed with Primer Premier 5.0. The reaction was performed in a Hard-Shell^®^ High-Profile 96-well Semi-Skirted PCR plate (Bio-Rad, United States). The amplification protocol was as follows: 95°C for 5 min, then 40 cycles of 95°C for 10 s, 57°C for 20 s, and 72°C for 20 s, with a final cycle of 95°C for 15 s, 60°C for 60 s, 95°C for 15 s, as for melt curve analysis. The *β-actin* gene was chosen as a control for normalization of the qRT-PCR (Salem et al., 2006). The relative expression ratio (R) was calculated using the 2^−ΔΔ^*^C^*^t^ method (Livak and Schmittgen, 2001).

**Table 1:** PCR primers used in the study.

Gene name	Primer sequences	Product size
*β-actin*	F: TGAAATTGCCGCACTGGTTG R: CTGAGCCTCGTCACCAACAT	169 bp
*nrf2*	F: CGCTAACGCAAACCAACACA R: GGAGCTGCATGCATTCATCG	102 bp
*keap1*	F: TTCCACGCCCTCCTCAA R: TGTACCCTCCCGCTATG	210 bp
*hsp70*	F: CGTGGTGTTCCCCAGATTGA R: CGCTGCACATCATCTTCAGC	194 bp
*hsp90*	F: CAGAGGCACCACCATTACGT R: AATGGGCTCCTCTACGGTCT	154 bp
*chip*	F: ACTGTAAACACGCCCTCGAG R: CGTCTCCAAAATTCAGCCGC	156 bp
*ub*	F: GCCAAGCGACACCATTGAG R: GGATGTTGTAGTCGGACAG	150 bp
*psma2*	F: CAGGCCAGCTTGTTCAGAGA R: TCCCAGCCAGCAATCAGAAG	100bp
*psmc1*	F: CGGCTGCTCTGTGTTACTGA R: ACTCTGGATGTGTGAGGGGA	191 bp
*murf1*	F: TGTCTATGGACTACAGAGGAA R: GGATTTCAAAGGAGGTTCAAG	103 bp
*mafbx*	F: CGGACGAGATCTGGTTAGCC R: GCTTGCGGATCTGTCTGTCT	119bp
*s6k1*	F: TGCCAACTGTTGCCTCGTAA R: AAAGCCACTGCACTGATCCA	166 bp
*mtor*	F: CGAGAAGGGTTTTGATGA R: GATGTGACGAGGCTTAGTG	217 bp

### Dot-Blotting

Total protein was isolated from muscle by RIPA Lysis Buffer (GBCBIO Technologies, China), and the concentration was determined with BCA Protein Assay Kit (GBCBIO Technologies, China). The concentrations of samples should be held same by PBS (0.67 μg μL^−1^). Then, the proteins were loaded on the Nitrocellulose Membrane (PALL Bio Trace^TM^ NT) through the template of Bio-Dot^®^ (Bio-Rad, United States). The membrane was kept in Blocking Buffer for 1 h to block nonspecific binding, after that, it was incubated overnight at 4°C with the primary antibody (diluted 1:1000). MAb to Polyubiquitinylated Conjugates (FK1) (Biomol, BML PW8805-0500) had been shown to recognize polyubiquitinylated proteins. After incubation with the secondary antibody which is labeled by FITC (anti-mouse, BOSTER, BA1101), the membrane was observed by Odyssey CLx (LI-COR, CLx-0813). We used ImageJ to compute the levels of ubiquitinated proteins.

### Tyrosine and 3-Methylhistidine Determination

The 3-MH provides a reliable index of the rate of myofibrillar protein breakdown in the musculature (Yong and Munro, 1978). Tyrosine is present in all myoblast, indirectly reflecting the degradation of total protein in the muscle. The contents of the free (3-MH) and Tyrosine (Tyr) of muscle were determined by High Performance Liquid Chromatography (HPLC) according to the National Standard of the People’s Republic of China (GB/T5009.124-2003).

### Data and Statistical Analysis

All data were determined with one-way analysis of variance (ANOVA) following by Tukey’s HSD test with SPSS software. The differences were deemed statistically significant at *P* < 0.05.

## Results

### Fish Growth

Final weight (FW), final length (FL), weight gain rate (WGR), specific growth rate (SGR), feed conversion efficiency (FCR), and feed ration (FR) in each group are shown in **Table 2**. Compared to the HSD group, fish exhibit significantly higher growth rate in the LSD group (*P* < 0.05).

**Table 2:** Growth parameters of the grass carp kept at low (LSD, 0.9 kg m^−2^) or high (HSD, 5.9 kg m^−2^) stocking density for a period of 70 days.

	LSD	HSD
Initial weight (g)	96.80 ± 18.56	99.56 ± 15.16
Initial length (cm)	17.72 ± 0.63	17.64 ± 0.27
Final weight (g)	206.01 ± 44.57	161.42 ± 4.42^∗^
Final length (cm)	22.17 ± 1.38	20.82 ± 0.22^∗^
Weight gain rate	112.82 ± 21.48	62.13 ± 8.27^∗^
Specific growth rate	1.15 ± 0.16	0.81 ± 0.08^∗^
Feed conversion efficiency	1.61 ± 0.04	1.61 ± 0.02
Feed ration	1.57 ± 0.16	1.14 ± 0.11^∗^

*After the experimental period, the final weight (FW), final length (FL), weight gain rate (WGR), specific growth rate (SGR), feed conversion efficiency (FCR), and feed ration (FR) were measured. Data are expressed as mean ± SD (n = 8). Significant differences were indicated with an asterisk at P < 0.05.*

### Stress-Related Parameters

**FIGURE 2:**
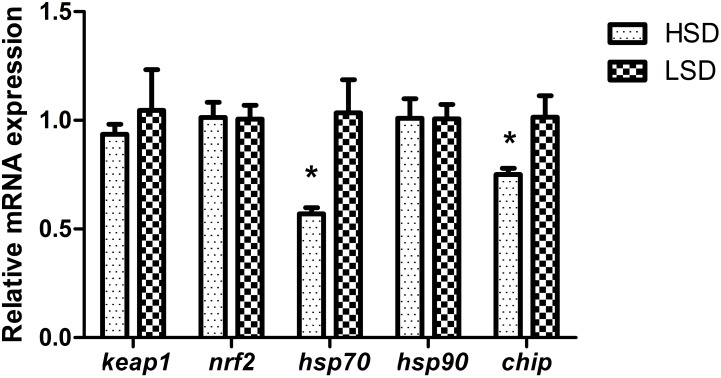
Expressions of *keap1*, *nrf2*, *hsp70*, *hsp90*, and *chip* in the white muscle of grass carp. Fish was randomly reared at low stocking density (LSD, 0.9 kg m^−2^) or high stocking density (HSD, 5.9 kg m^−2^) for 70 days, dorsal white muscle (5 individuals per tank) were collected for analysis. The expression in two densities with the expression level in LSD as 1. Significant differences were indicated with an asterisk at *P* < 0.05. Data are shown as means ± SEM (*n* = 4).

There were no significant differences in serum levels of cortisol, TP and GLU between two groups (**Table 3**). *Nrf2* and *keap1* mRNA levels were not obvious differences between HSD and LSD. HSD did not cause a significantly increased in the expression of *hsp90*. The expression levels of *hsp70* and *chip* in HSD group were significantly lower than those in LSD group (*P* < 0.05, **Figure 2**).

**Table 3:** Serum biochemical parameters of the grass carp kept at low (LSD, 0.9 kg m^−2^) or high (HSD, 5.9 kg m^−2^) stocking density for a period of 70 days.

	LSD	HSD
Glucose (GLU) (U L^−1^)	6.04 ± 0.91	4.54 ± 0.99
Total protein (TP) (g L^−1^)	36.33 ± 6.31	32.82 ± 5.47
Cortisol (mmol L^−1^)	146.87 ± 48.71	152.1 ± 67.68

*Serum Cortisol, glucose (GLU), and total protein (TP) was analyzed at the end of the experiment. Data are expressed as mean ± SD (n = 8).*

### The Expression Levels of UPS-Related Genes and Ubiquitinated Proteins

**FIGURE 3:**
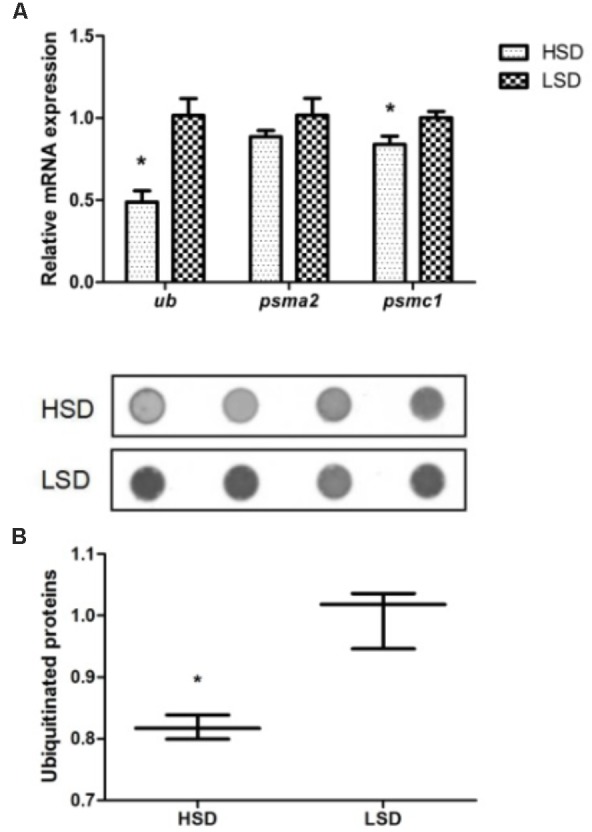
Expressions of *ub*, *psma2*, *psmc1*, and the ubiquitinated proteins in the white muscle of grass carp. **(A)** The mRNA expressions of *ub*, *psma2*, and *psmc1.* **(B)** Dot blot analysis about the ubiquitinated proteins. Dorsal white muscle (5 individuals per tank) were collected after the experimental period of 70 days. The expression in two densities with the expression level in LSD as 1. Significant differences were indicated with an asterisk at *P* < 0.05. Data are shown as means ± SEM (*n* = 4).

The expression levels of *ubiquitin* (*ub*) and *psmc1* were significantly declined in the HSD group compared to the LSD group (*P* < 0.05, **Figure 3A**). The expression of *psma2* was not significant difference in two groups. A significant high content of ubiquitinated proteins occurred in white muscle of fish in LSD group (*P* < 0.05, **Figure 3B**).

### The Parameters Related to Protein Metabolism

**FIGURE 4:**
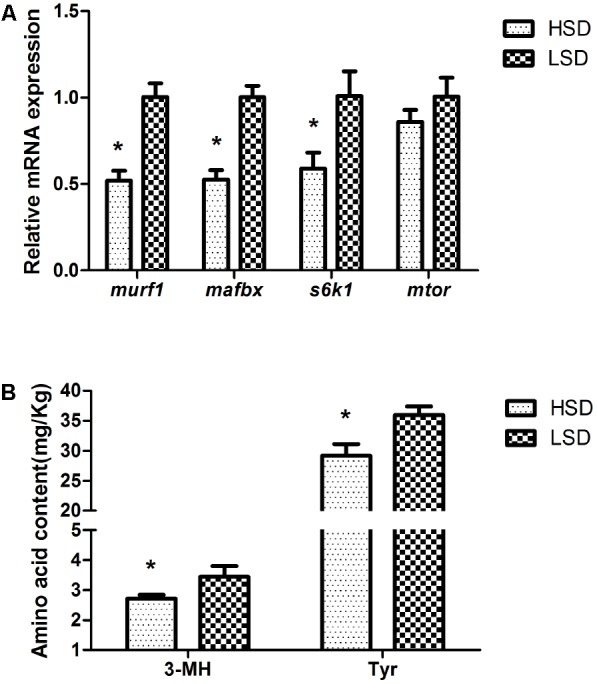
Expressions of *murf1*, *mafbx*, *s6k1*, *mtor*, and contents of 3-MH and Tyr in the white muscle of grass carp. **(A)** The mRNA expressions of *murf1*, *mafbx*, *s6k1*, and *mtor.* **(B)** Amino acid contents of 3-MH and Tyr. Dorsal white muscle (5 individuals per tank) were collected after the experimental period of 70 days. The expression in two densities with the expression level in LSD as 1. Significant differences were indicated with an asterisk at *P* < 0.05. Data are shown as means ± SEM (*n* = 4).

The mRNA levels of *murf1*, *mafbx*, and *s6k1* decreased significantly in the HSD group (*P* < 0.05, **Figure 4A**). On the other hand, the stocking density did not modify the expression of *mtor*. The contents of 3-MH and Tyr were significantly higher in the LSD group than those in the HSD group (*P* < 0.05, **Figure 4B**).

## Discussion

Growth is a good indicator of fish health. Rapid growth usually reflects abundant food supply and appropriate environmental conditions. The present study found that high stocking density had a negative effect on growth performance of grass carp. The similar findings have been reported in many fish species, such as *Salminus brasiliensis*, *Megalobrama amblycephala*, and *Acipenser schrenckii* (Braun et al., 2010; Li et al., 2012; Qi et al., 2016). Generally, the levels of serum cortisol and glucose as well as the expressions of *hsp70*, *hsp90*, and *keap1*-*nrf2* signaling pathway can indicate the magnitude of fish stress (Lushchak, 2011). In this study, however, significant difference in the levels of either cortisol or glucose between groups was not found. The similar results can be observed in other fish species (Tort et al., 1996; Rotllant et al., 1997). In fish, *nrf2* is a critical factor that regulates the expression of a group of genes encoding antioxidant enzymes (Lushchak, 2011). Under the salinity exposure, the gene expression of *nrf2* in mosquito fish (*Gambusia affini*s) showed an obvious dose-effect (Bao et al., 2017). Nevertheless, not only the serum biochemical parameters levels but also the mRNA abundances of *keap1*, *nrf2*, and *hsp90* did not change significantly between two groups at the end of the experiment in this study. Heat shock proteins are generally considered as characteristic biomarkers of cellular stress responses and contribute to numerous cellular signaling pathways (Benarroch, 2011; Kirschke et al., 2014). It indicates that denatured proteins accumulate upon exposure to stressors (Benarroch, 2011). In rainbow trout the mRNA levels of *hsp70* remained at similar levels (Liu S. et al., 2014), and in senegalese sole (*Solea senegalensis*) it decreased after exposure to high stocking density (Salas-Leiton et al., 2010). In this study, the expression of *hsp70* increased in the LSD group. A reason for the variable expression of *hsp70* may be attributed to their pleiotropic functional spectra (Kirschke et al., 2014). The plasma cortisol concentration often surged in the early stage of stress, and then returned to the control after adaptation with a long-term (Rebl et al., 2017). No significant difference in stress-related parameters between two stocking density groups probably be associated with the duration of crowding stress. Grass carp in the HSD group might adapt to the crowding environment after 70-day culture period.

Proteolysis by the UPS is ATP-dependent, and Ub, E1, E2, E3, and 26S proteasome are required in this process. 26S proteasome is formed by 19S regulatory ATPase complex and 20S proteasome complex (Todgham et al., 2017). The stocking density did not affect the *psma2* expression, while the higher mRNA levels of *ub* and *psmc1* were observed in the LSD group, coupled with the elevation of the content of ubiquitinated proteins. This suggests strong activity of UPS in fish stocked at lower density. It has been demonstrated that the activity of the UPS was enhanced during the process of expenditure and atrophy of skeletal muscle (Dobly et al., 2004). And high expression of *psmc1*, *mafbx*, and *murf1* appeared in the muscle of juvenile fine flounder (*Paralichthys adspersus*) at 7 weeks of crowding (Valenzuela et al., 2017).

E3 ligases, MuRF1 and MAFbx are regarded as the primary markers in the process of protein degradation (Bodine et al., 2001), and the enhanced expression of *mafbx* and *murf1* activated the protein breakdown in mice (Choi et al., 2017). Actin and myosin are the contractile proteins of skeletal muscle, and 3-Methylhistidine (3-MH) can be detected in hydrolysates of these proteins (Yong and Munro, 1978). Consequently, the higher contents of 3-MH and Tyr coupled with higher mRNA abundances of *mafbx* and *murf1* indicated more degraded proteins in fish muscle of the LSD group. Although more protein degradation occurred in white muscle, fish grew better in the LSD group. This means that synthetic metabolism is greater than catabolism. The tissues, such as heart, liver, and red muscle which contain relatively high abundance of proteins, showed high levels of *mafbx*. Furthermore, the relatively high level of metabolic activity of the tissues suggested that *mafbx* may influence the recycling of proteins involved in energy production or metabolic maintenance (Cleveland and Evenhuis, 2010).

The activation of mTOR positively mediates protein synthesis through the phosphorylation of S6K1, which can promote ribosomal protein expression and cell growth (Howell et al., 2013; Huynh et al., 2017). The high expressions of *s6k1* and *mtor* in the LSD group suggest the activation of the protein synthesis in fish muscle. And *hsp70* and *chip* also showed apparently high expression in the LSD group. In addition to a presumptive role in re-folding thermally damaged proteins, *hsp70* seems to be part of a molecular chaperone complex. Hsp70 binds to polypeptides translating on ribosomes and regulates the protein biogenesis under normal cellular conditions (Frydman et al., 1994). Therefore, fish in the LSD group showed a high rate of protein synthesis. Previous research demonstrated that the expression of MAFbx, MuRF1, and mTOR were significantly increased in the IGF-1 treatment duck (*Anas platyrhynchos*) (Liu H.H. et al., 2014). It is inferred that IGF-1 can promote animal growth. The growing rats feed with a zein protein diet showed the increased expressions of mTOR, MAFbx, and MuRF1, and high protein metabolism (Luo et al., 2010). Therefore, not only protein synthesis, but also protein degradation manifested high activity in LSD.

## Conclusion

The results demonstrated stocking density exerted potent effects on growth performance and UPS activity in muscle of grass carp. A greater growth rate occurred in the fish cultured at lower stocking density. The fish with a better growth performance exhibited a higher UPS activity in parallel with the activation of protein synthesis. These results illustrate that the fish cultured at low stocking density would exhibit a greater growth rate and a fast protein turnover in muscle.

## Author Contributions

YS and XL performed the experiment, analyzed the data, and wrote the manuscript. DL designed the experiment and polished the manuscript. JC cultured the fish and analyzed the growth data. RT and LL revised the manuscript. All authors reviewed the manuscript.

## Conflict of Interest Statement

The authors declare that the research was conducted in the absence of any commercial or financial relationships that could be construed as a potential conflict of interest.
